# Nature and Specificity of Gestural Disorder in Children with Developmental Coordination Disorder: A Multiple Case Study

**DOI:** 10.3389/fpsyg.2017.00995

**Published:** 2017-07-04

**Authors:** Orianne Costini, Arnaud Roy, Chrystelle Remigereau, Sylvane Faure, Catherine Fossoud, Didier Le Gall

**Affiliations:** ^1^Unité Vision & Cognition, Fondation Ophtalmologique Adolphe de RothschildParis, France; ^2^Laboratoire Psychologie de la Perception, CNRS UMR 8242, Centre Biomédical des Saints-Pères, Université Paris DescartesParis, France; ^3^Laboratoire de Psychologie des Pays de la Loire, EA4638, Université Nantes Angers Le MansNantes, France; ^4^Centre de Référent des Troubles d’Apprentissage, Hôpital Mère-Enfant, Centre Hospitalier Universitaire de NantesNantes, France; ^5^Centre de Compétence Nantais de Neurofibromatose, Hôpital Hôtel-Dieu, Centre Hospitalier Universitaire de NantesNantes, France; ^6^Laboratoire d’Anthropologie et de Psychologie Cognitives et Sociales, EA 7278, Université Nice Sophia AntipolisNice, France; ^7^Centre de Référence des Troubles des Apprentissages, Hôpitaux Pédiatriques de Nice CHU-LenvalNice, France

**Keywords:** developmental coordination disorder, dyspraxia, gestures, praxis, child

## Abstract

**Aim:** Praxis assessment in children with developmental coordination disorder (DCD) is usually based on tests of adult apraxia, by comparing across types of gestures and input modalities. However, the cognitive models of adult praxis processing are rarely used in a comprehensive and critical interpretation. These models generally involve two systems: a conceptual system and a production system. Heterogeneity of deficits is consistently reported in DCD, involving other cognitive skills such as executive or visual-perceptual and visuospatial functions. Surprisingly, few researches examined the impact of these functions in gestural production. Our study aimed at discussing the nature and specificity of the gestural deficit in DCD using a multiple case study approach.

**Method:** Tasks were selected and adapted from protocols proposed in adult apraxia, in order to enable a comprehensive assessment of gestures. This included conceptual tasks (knowledge about tool functions and actions; recognition of gestures), representational (transitive, intransitive), and non-representational gestures (imitation of meaningless postures). We realized an additional assessment of constructional abilities and other cognitive domains (executive functions, visual-perceptual and visuospatial functions). Data from 27 patients diagnosed with DCD were collected. Neuropsychological profiles were classified using an inferential clinical analysis based on the modified *t*-test, by comparison with 100 typically developing children divided into five age groups (from 7 to 13 years old).

**Results:** Among the 27 DCD patients, we first classified profiles that are characterized by impairment in tasks assessing perceptual visual or visuospatial skills (*n* = 8). Patients with a weakness in executive functions (*n* = 6) were then identified, followed by those with an impaired performance in conceptual knowledge tasks (*n* = 4). Among the nine remaining patients, six could be classified as having a visual spatial/visual constructional dyspraxia. Gestural production deficits were variable between and within profiles.

**Discussion:** This study confirmed the heterogeneity of gestural production deficit among children with a diagnosis of DCD, at both intra- and inter-individual levels. The contribution of other cognitive deficits in most of the profiles allows discussing the specificity of gestural difficulties. This argues in favor of the necessity to distinguish gestural problems with other deficits made apparent through gesture.

## Introduction

Since its identification many years ago, the concept of a specific developmental disorder of motor function raises many debates in clinical practice as well as in literature. The two major clinical diagnoses are developmental coordination disorder (DCD) and developmental dyspraxia. According to the updated DSM criteria (DSM-V, American Psychiatric Association [APA], 2013), DCD is defined by (1) a marked impairment in the acquisition and execution of coordinated motor skills given the child’s chronological age and opportunity for skill learning and use, (2) which has a significant and persistent impact on daily living activities or academic achievement, (3) with symptoms that were present since the early developmental period, (4) when there is neither intellectual disability, nor visual impairment, nor a neurological condition that would better explain the disturbance. Developmental dyspraxia refers more specifically to “praxis.” It relates to the ability to plan and perform skilled and purposeful motor actions or movement sequences, and the ability to use tools (Goodgold-Edwards and Cermak, 1990; Poole et al., 1997; Dewey et al., 2007). By contrast with apraxia, developmental dyspraxia is not an acquired disorder. Rather, it refers to a constitutional and idiopathic disorder occurring in a developmental context (Vaivre-Douret, 2014). While a distinction has been postulated by some authors (Miyahara and Möbs, 1995; Steinman et al., 2010), dyspraxia is often considered to be part or a synonymous of DCD. Successive consensus meetings validated the term “DCD,” inviting researchers to use this diagnosis to describe all children who exhibit motor coordination problems. However, the theoretical definition and diagnosis of DCD remain unclear, making it heterogeneous and non-specific (Blank et al., 2012). Attempts at classification have failed to identify consensual subtypes of DCD (see Vaivre-Douret, 2014, for review). Despite this, it is worth noting that clinical research and practice still refer to apraxia subtypes using the terms “ideomotor,” “ideational,” and “constructive” (Njiokiktjien et al., 2000; May-Benson and Cermak, 2007; Vaivre-Douret et al., 2011a,b). Furthermore, studies about typical and atypical development of gestures are based on tasks directly transposed from the clinical tests used on adults and thus referring to the cognitive models of apraxia.

### Cognitive Models of Apraxia

These models classically distinguish a conceptual system and a production system (Osiurak et al., 2011; Osiurak and Le Gall, 2012). The conceptual system generally includes different kinds of knowledge, supporting the formation of a mental representation of the action. Firstly, the semantic knowledge about tool function, which contains information that concerns the conventional use (that is, for which action, associated object actions, in which context) (Rothi et al., 1991; Roy, 1996; Buxbaum, 2001). Roy and Square (1985), whose model was recently updated by Stamenova et al. (2012), also proposed the existence of semantic knowledge about action. It specifies body movements that characterize an action independently of the usually associated tool (decontextualized). Secondly, the assumption of sensorimotor knowledge about usual tool manipulation (or “gestural engram”) was proposed. It is defined as stored representations of familiar gestures, containing critical and invariant sensorimotor characteristics specific for an action (usual tool manipulation) according to an effector (Rothi et al., 1991; Buxbaum, 2001; Buxbaum and Kalénine, 2010). The production system supports the effective execution of gestures (familiar or new) (Roy and Square, 1985; Rothi et al., 1991; Buxbaum, 2001). As a dynamic system, it allows the generation of a pattern of movements adapted to the environmental constraints (spatial and temporal), coordinated as part of an egocentric reference frame (body-centered), according to the gestural representation at the conceptual level (Buxbaum, 2001). At this level, a perceptual-motor process would enable the organization and the execution of the action (Roy and Square, 1985; Zoia et al., 2002).

On the basis of clinical dissociations, the above cognitive models of apraxia suggested that there are multiple “routes” to action. Two routes would underlie the transitive gestures (involving the use of tool): a verbal route and a visual route (Rothi et al., 1991; Rumiati and Humphreys, 1998). More specifically, these transitive gestures could be generated via the access to semantic knowledge (“semantic route”), or by a direct association between a structural analysis of the visually presented tool, and the stored sensorimotor knowledge relating to the associated action (“non-semantic route”) (Rothi et al., 1991; Buxbaum, 2001). It is worth noting that possible compensations between these two routes were suggested. In addition, a “non-lexical route” would allow the processing of unrecognized, new, or meaningless gestures (Rothi et al., 1991; Bartolo et al., 2001). According to Rothi et al. (1991), this route supports a direct transformation of the perceived visuo-gestural information into motor patterns (“direct route”). Nevertheless, doubt was raised about the existence of this direct route due to the observed dissociations between imitation of unfamiliar finger postures (reproduction under direct visual control) and hand postures (hand applied to the face without visual control). Indeed, finger postures would depend on a visuospatial processing while hand postures would require a body-part coding related to knowledge about the structure of the human body (Goldenberg, 2001).

### Praxis Examination in DCD Children

Praxic skills in children are mainly examined through the production of representational limb gestures that are transitive (requiring the use of an imagined tool) or intransitive (symbolic, communicative gestures), according to a variety of commands (e.g., verbal; **Table 1**). DCD children generally performed lower than their typically developing peers on representational gestures (Dewey, 1991, 1993; Dewey and Kaplan, 1992; Hill, 1998; Zoia et al., 2002; Sinani et al., 2011). Representational transitive gestures are less well-performed than intransitive gestures, with a greater disruption under verbal command than imitation (Dewey, 1991, 1993; Dewey and Kaplan, 1992; Hill, 1998). These results could suggest difficulties related to the knowledge of gestures, but few studies examined this knowledge without any production (**Table 1**). Nonetheless, some studies have ruled out a semantic (conceptual) deficit (Hill et al., 1998; Sinani et al., 2011). Concerning the visual-gestural route, Dewey (1991) showed equivalent performances in the recognition of familiar representational gestures between patients and controls. Sinani et al. (2011) found no significant difference in a task evaluating the knowledge about prototypical tool manipulation (the child had to visually identify the correct use of an object illustrated on four pictures, in which only one illustrated the correct action). These authors therefore suggested that impaired ability of DCD children to produce familiar gestures was not due to conceptual problem. Surprisingly, they still argued for the presence of an impairment relating to the sensorimotor representation of previously known actions. This assumption was based on the performance of DCD children on the “Praxis Imagery Questionnaire,” with difficulties mainly concerning questions about hands/fingers position, and limb movements while executing the action (imagined). No significant difference was reported for questions about joints movement, and the physical properties of the tool used in the action. It has to be noted here that the supposed mobilization of the sensorimotor representation of the familiar transitive representational gesture is executed under verbal command, therefore by verbal route.

**Table 1:** Praxis assessment (upper limbs) according to studies.

Studies	Knowledge	Representational Gestures		Meaningless Gestures	Tool Use
Dewey (1991)	RG REC	T IT	VC, IM VC, IM	*NA*	Single
Dewey and Kaplan (1992)	*NA*	T IT	VC, IM VC, IM	Postures (IM)	*NA*
Dewey (1993)	*NA*	T, IT	VC, IM VC, IM	*NA*	*NA*
Poole et al. (1997)	*NA*	T IT	VC, IM VC, IM	*NA*	*NA*
Hill (1998)	*NA*	T IT	VC, IM VC, IM	Postures (IR, IM) Sequences (IR, IM)	*NA*
Hill et al. (1998)	*NA*	T IT	VC, IM VC, IM	*NA*	*NA*
Zoia et al. (2002)	*NA*	T	VC, IM, VP	*NA*	Single
Dewey et al. (2007)	*NA*	T IT	VC, IM VC, IM	*NA*	*NA*
Vaivre-Douret et al. (2011b)	*NA*	T	VC	Postures (IM) Sequences (IM)	*NA*
Sinani et al. (2011)	PIQ REC of Use	T IT	VC, VC/eyes closed, IM VC, VC/eyes closed, IM	*NA*	Single Single/eyes closed
Giofrè et al. (2014)	*NA*	IT	VC, IM	Postures (IM, VC)	*NA*

*RG: representational gestures; NA: not assessed; REC: recognition; T: transitive gestures; IT: intransitive gestures; VC: verbal command; IM: imitation; IR: immediate recall; VP: visual presentation of the tool; PIQ; Praxis Imagery Questionnaire (Sinani et al., 2011). In a Single tool use task, the child is asked to demonstrate the use of a tool while holding it in hand, but without the usual corresponding object (for further explanations about the different ways of assessing tool use, see Baumard et al., 2014).*

In DCD children, the hypothesis of an impairment of the sensorimotor representation of the gesture raises several questions, in particular about the nature of information stored in memory. This representation evidently rests on sensorimotor knowledge hypothesis (“gesture engrams”) found in the literature on apraxia, even if its features are not clearly defined (Zoia et al., 2002). The results of DCD children detailed above suggest that the mobilization of this representation would be more specifically impaired in verbal command modality than in visual/visuo-gesture, whether the task implies an effective production or not. The contribution of poor verbal comprehension or lack of familiarity was excluded by the authors (Hill, 1998; Sinani et al., 2011). The rare studies proposing a condition of use (object in hand), except for Zoia et al. (2002), showed a significant improvement or a normalization of DCD children performances (Dewey, 1991; Sinani et al., 2011). If the difficulties of these children are related to the formation and the recall of the sensorimotor representation of gestures (Zoia et al., 2002; Sinani et al., 2011), how can an object provide sufficient cues to correctly perform the action? And in this case, why is the difficulty to use tool in everyday life activities one of the main complaints of DCD children? Also, how can impairment of meaningless gestures (under imitation) observed in some studies be explained (Dewey and Kaplan, 1992; Vaivre-Douret et al., 2011a,b)? Indeed, those gestures are not referring to any stored gestural representation (production system alone), because they are new and unknown (Vanvuchelen et al., 2011). Note that this type of gesture constitutes the material of the only normalized clinical tests (Test of imitation of gestures of Bergès and Lézine, 1963; NEPSY Imitation of hand positions, Korkman et al., 2003). The underlying cognitive processes of the praxic deficits observed in DCD children thus remain to be clearly specified. Considering that the assessment of praxic skills in children uses the clinical tools from apraxia, the absence of explicit confrontation to cognitive models that established the validation of these tools represents a fundamental limit.

Beyond the fact that the examination is often limited to representational gesture (**Table 1**), most of studies on gestural performances in DCD children neglected the possible intervention of other cognitive skills in the motor development (Zoia et al., 2004). Vaivre-Douret et al. (2011a,b) emphasized the need for a complementary investigation of perceptive skills and executive functions. Indeed, the impairment of those skills might have an impact on gestural performance, which should be distinguished from specific praxic disorders. Considering these aspects is fully justified in DCD, since executive deficits were evoked in this syndrome (Wilson et al., 2012; Toussaint-Thorin et al., 2013; Pratt et al., 2014) as well as visuoperceptive and visuospatial disturbances (Tsai et al., 2008; Rösblad, 2011; Wilson et al., 2012). In the same way, whereas the lack of consensus on underlying deficits and sub-types identification (Wilson et al., 2012; Vaivre-Douret, 2014), the literature regularly highlights the wide inter- and intra-individual variability of disorders in DCD (King et al., 2011). Thus, if general linear statistical models (e.g., ANOVA) allow distinguishing DCD groups from those with typical development on some measures (King et al., 2011), they do not allow the characterization of individual differences in performance. A better comprehension of these differences is, however, essential for the evaluation of the predictions from cognitive models. In this respect, the interest of case series studies has been emphasized (Rapp, 2011).

### The Present Study

The first aim of the study is to analyze the nature and specificity of gestural disorders in children with DCD. In the absence of developmental models about praxic skills, the protocol was designed “theoretically” in reference to cognitive models of gestural processing in adult, more particularly those of Roy and Square (1985) and Rothi et al. (1991). Despite the obvious limitations of this method, it represents an essential step in the progress of knowledge about atypical development of praxis in children. Considering the heterogeneity of the deficits in DCD, and in order to be able to confront the results with theoretical models predictions, this research is designed according to a multiple case method. The objective was to document at the individual level (1) the efficiency of the different processing levels that are distinguished by cognitive models using an exhaustive children’s adapted protocol, (2) the contribution of other cognitive functions to performances, as evoked by the literature in DCD (verbal comprehension, visual perception, visuospatial processing, executive functions). The proposed hypothesis is that children with DCD are mainly impaired in their production of gestures, in the absence of specific deficit on semantic or sensorimotor knowledge. On the other hand, considering the demonstrated heterogeneity of deficits in DCD, it is expected that gestural disturbances would be observed as well as other cognitive impairment non-specific to the gesture.

## Materials and Methods

### Participants

The clinical population was selected on the basis of retrospective analysis of the medical records of children referred to reference centers for learning disabilities of university hospitals of Nice and Nantes (consultation or follow-up). School children aged 7–13 years old and diagnosed to have developmental dyspraxia^1^ or DCD on the basis of a multidisciplinary assessment fulfilling DSM-IV-R were included in the study. The following extended exclusion criteria were used for this study: non-native French speakers, unknown medical history (e.g., adopted child), neurological disease, pervasive developmental disorder/autism spectrum disorder, mental disorders, premature birth, intellectual disability, sensory deficit or verbal comprehension disorder that may interfere with the administration of tests. Thirty-nine patients with DCD were enrolled in this study. Nine of them later appeared to meet one of the described exclusion criteria, and important data were missing for 3 others. So, the study comprised 27 patients.

Considering the experimental nature of the praxis tasks, a sample of typically developing children was constituted to allow comparison of a patient’s test score to a control group. These children were recruited from schools with the permission of the relevant authority. Exclusion criteria (common to the DCD patients) were verified thanks to an anamnestic questionnaire. The absence of proven or suspected neurodevelopmental disorders was also checked. Since the control children were not assessed on the full Wechsler scale, the present study included Matrix Reasoning and Vocabulary subtests (WISC-IV; Wechsler, 2005) because they are highly related to factor G (Total IQ). Handedness quotient was calculated (with a value of +100 representing extreme right hand preference, according to the Edinburgh Handedness Inventory; Oldfield, 1971). Socio-economic status (SES) was also registered for each child. It was indicated by mean educational level of the two parents, expressed in years of schooling. The control group included 100 children, distributed according to five age groups: 7, 8, 9, 10, and 11–13 years. Except for gender, no significant difference was found between the various age groups on the demographic and controlled variables (**Table 2**). Note that none of the 27 patients showed a significantly different SES, by comparison to the appropriate age group (according to one-tailed Student’s test, with threshold *p* < 0.05, as described below).

**Table 2:** Demographic characteristics and controlled variables for each age groups.

Group	7 years	8 years	9 years	10 years	11–13 years	*F*	*p*
**TD**							
*n*	20	19	20	20	21		
Sex	15G/5M	12G/7B	6G/14B	10G/10B	8G/13B	2.84	0.03
SES	14.15 (4.34)	11.82 (3.76)	12.10 (5.24)	13.02 (3.06)	12.83 (3.61)	0.99	0.42
LQ	89.28 (15.44)	83.42 (27.96)	73.25 (51.50)	52.84 (68.99)	51.36 (72.12)	2.14	0.08
Mat. SS	11.70 (2.56)	10.79 (3.41)	11.80 (2.89)	10.95 (2.09)	11.24 (2.61)	0.05	0.72
Voc. SS	13.40 (2.48)	11.95 (2.44)	11.65 (2.46)	11.75 (2.31)	13.05 (2.38)	2.25	0.07

*TD, typical development; G, girls; B, boys; SES, socio-economic status; LQ, laterality quotient calculated with Edinburgh inventory (Oldfield, 1971); Mat. SS, scaled score obtained on the Matrix Reasoning subtest of WISC-IV; Voc. SS, scaled score obtained on the Vocabulary subtest of WISC-IV.*

Participants were enrolled between March 2010 and January 2013. The research was conducted in accordance with the University guidelines and the ethical standards established by the Declaration of Helsinki. All children and their parents were provided with an information note detailing the procedure of the study. Informed consents were obtained from children and parents before carrying out the assessment.

### Material and Procedure

#### Assessment of Praxis Functioning

As noticed by Zoia et al. (2002), praxis assessment in children is commonly based on the examination of apraxia, but no battery has been consensually agreed for assessing gestures in children. Therefore, we developed a protocol of praxis assessment for children, based on protocols proposed in adults by Le Gall et al. (2000), and Peigneux and Van der Linden (2000). Tasks were selected in order to comprehensively assess the different levels of gestural organization predicted by the previously mentioned theoretical models of apraxia (**Table 3**). The present protocol was used in part for assessing gestural difficulties in children with Neurofibromatosis type 1 (Remigereau et al., 2017).

**Table 3:** Assessed tests and components.

	Semantic knowledge	Sensorimotor knowledge	Motor production
**Conceptual tasks (KNOW)**			
Naming tools by function	+	–	–
Functional association PEGV	+	–	–
Knowledge of actions	+	–	–
Matching pantomime-tool	–	+	–
**Representational gestures**			
Pantomimes – verbal instruction	+	+	+
Pantomimes – visual instruction	+	+	+
Symbolic gestures (intransitive)	+	+/-	+
**Imitation of meaningless postures**			
Finger configuration	–	–	+
One-hand configuration	–	–	+
Two-hands configuration	–	–	+
**Imitation of meaningless sequences**			
Finger sequencing task	–	–	+
Manual motor sequences NEPSY	–	–	+
**Real tool use**			
Multiple choice task	+/-	+/-	+
Multiple object task	+/-	+/-	+

*+, strong involvement; +/-, moderate involvement; –, weak or no involvement.*

All children complete the different tasks in the same order. The administration of tests was carried out on two individual settings separated with a maximum interval of 15 days. The length of the sessions was around 1.5–2 h in total (only for the praxis protocol). Performances on gestural productions were videotaped for later scoring by the investigator. The first author was in charge of all the scoring.

##### Conceptual tasks

Semantic knowledge about tool functions was assessed by means of two tasks.

On one task (*Naming tools by function*), the children had to name the 12 tools used in the pantomime tasks after a verbal description of their usual function. The *Functional association* subtest of the PEGV (Visual Gnosia Evaluation Protocole; Agniel et al., 1992) was also used. In this task initially thought to assess the associative visual agnosia, the child was presented with two distractors and asked to identify the drawing of the tool associated with the proposed target tool (e.g., envelope – stamp).

*Knowledge of actions* was assessed by asking the child to find the tool allowing for the execution of a specific action, the usual tool being non-presented. The target (functional equivalent) and the three different types of distractor objects (motor, semantic, and neutral) were presented on pictures.

Finally, the ability to recognize transitive gestures was examined with a task of *Matching pantomime-tool*. The child was asked to point to the picture of the tool that matches the pantomime carried out by the experimenter (six pantomime items). The proposed pictures included three different types of distractors (motor, semantic, and neutral).

For each one of these tasks, every correct answer was awarded one point.

##### Representational gestures

Twelve transitive gestures (*pantomimes*) and twelve intransitive gestures (*symbolic gestures*) were proposed. Both types of gestures were subdivided into six reflexive (toward the body) and six non-reflexive items. The transitive and intransitive gestures were performed under verbal command (starting by tool naming) in the first setting. In the second one, transitive gestures were performed under visual command (presentation of the tool’s picture).

The child carried out the various gestures while sitting opposite the investigator, first with his preferred hand (spontaneously used), then with the other. Each task was introduced with instructions followed by an example item, for which a feedback was provided to the child in order to make sure of his good comprehension of the task constraints. For each assessment item, a second trial was allowed if necessary. Every gesture was scored as two if performed correctly (without error) on the first trial, as 1 if performed correctly on the second trial, or 0 if it was performed incorrectly. A gesture was considered as incorrect if an error was made (content, temporal or spatial disruptions) or if it was unrecognizable or not executed at all.

##### Imitation of meaningless postures

Twenty-four meaningless postures (unknown gestures) were proposed. In order to facilitate the comparisons, each posture was matched with one of the representational gesture, according to criteria established by Peigneux and Van der Linden (2000): (1) configuration with finger prevalence (implying the fingers, independently of the position of the arm in space) or with hand (implying the hand and the arm, in a position related to the body), (2) one- or two hand- configuration, (3) static or dynamic property (implying movement), (4) overall complexity. According to the configuration type, postures were subdivided into three tasks (finger, one-hand, and two-hands configuration tasks), each of which including eight items.

In order to reduce memory and comprehension constraints, the child was asked to reproduce the posture demonstrated by the investigator in mirror and simultaneously (reciprocal imitation). The demonstrated posture was maintained until the child carried out his imitation, without time limitation. The three tasks were administered consecutively during the first session. The procedure and scoring were similar to those used for representational gestures.

##### Sequences of meaningless postures

A *Finger sequencing* task (thumb-index-thumb-ring) was carried out with each hand, starting with the preferred hand of the child. The task was stopped after nine executed sequences (correct or not). The score for each hand was determined by the number of sequences correctly performed.

The *Manual Motor Sequences* subtest of NEPSY was also administered, using its original rating system but no stop-point. This test has the advantage of including sequences using one or both hands but also sequential or simultaneous sequences. Each sequence had to be repeated five times by the child. The score corresponded to the number of correctly performed sequences.

For both tests, the sequence to be realized was demonstrated three times by the experimenter, in mirror. The example was repeated if required, and the assessment started only when the child has succeeded in performing alone the sequence (once).

##### Real tool use

Two tasks were proposed, asking the child to actually use tools with the corresponding object (usual). The score of each task corresponded to the number of correctly achieved actions with the corresponding usual tools.

In the *Multiple choice* task, the child was presented with several tools and objects on the table, some of which were not useful for the requested action (distractors). The child was then asked to perform a specific action by using a tool with the usual corresponding object. For each of the four actions, distractors either had functional, motor, morphological, or semantic similarities with the target-tool, or were neutral objects (unrelated).

In the *Multiple object* task, the child was asked to achieve three actions that required chronological steps and the handling of more than two objects. Only objects necessary to the action were given. It must be noted that this condition can be viewed as a choice condition, due to the presence of several objects that may be useful or not, according to the different steps of the action (Baumard et al., 2014).

#### Complementary Neuropsychological Assessment

Praxis examination of all children was completed by a neuropsychological assessment exploring cognitive domains shown to be involved in gestural performance. As previously mentioned, the *Matrix Reasoning* and *Vocabulary* subtests (WISC-IV; Wechsler, 2005) were proposed as they are highly correlated with the factor G (Total IQ). The *Comprehension* subtest of the NEPSY (Korkman et al., 2003) was used to evaluate verbal comprehension of instructions. Visual-perceptual functions were examined by means of the *Matching Geometrical Patterns* subtest (Visual Gnosia Evaluation Protocole; Agniel et al., 1992). In this task, a geometric shape was presented and the child had to identify the figure identical to the target among five distractors. The subtest *Arrows* (NEPSY) was used to assess visuospatial processing. Executive functions were also examined. To investigate verbal flexibility, inhibition, and planning, we used, respectively, the NEPSY’s subtest for verbal fluidity (named *Word Generation*), the *Stroop’s test* of Groupe de Réflexion sur l’Evaluation des Fonctions Exécutives [GREFEX] (2001), and the *2-part Rey-Osterrieth Complex Figure* (ROF) test as proposed by Roy et al. (2010). In this test, a traditional copy (“*Formulation*” condition) was proposed followed by a second copy (“*Execution*” condition) in which children progressively reproduced the figure on the basis of successive and progressive cues (see Roy et al., 2010 for cues and procedure). Each new group of elements was represented in a distinct color. The score for both conditions was the number of correct elements of the drawing in the correct locations, according to the traditional guidelines developed by Rey (1959). The difference of scores on cued and uncued ROF versions (planning index) was calculated by subtracting the Formulation condition score from the Execution condition score. This procedure was used to distinguish between children’s spontaneous planning ability (formulation condition, when structure is minimal) and their ability to execute an externally directed and highly structured strategy (execution condition). The latter condition should therefore provide a better measurement of visual-construction abilities, and was completed by *Block Design* subtest (WISC-IV).

### Data Analyses

The analysis of the clinical profiles of patients with DCD was conducted on the basis of the modified *t*-test method of Crawford and Howell (1998), which is more robust than Z score conversion (standard score) for testing the significance of the difference between an individual score and a standard derived from a small sample size (less than 50). Thus, the rough scores obtained for each of the 22 variables described above were compared with data from age distributions of the TD group. Performances were considered impaired when *p* < 0.05 according to one-tailed Student’s test. Given the ceiling effect associated with a low or zero variance in the tasks evaluating knowledge, real tool use, and Matching geometrical patterns (PEGV), performances were considered impaired when they were lower than the cut-off of the age of reference (the worst score achieved).

Results allowed identifying different groups on the basis of inferential clinical analysis, following a similar procedure to the one employed by Vaivre-Douret et al. (2011b). In order to address the specificity of gestural disturbances, the analysis identified clinical groups of patients with DCD who also had difficulties other than gestural. A procedure of progressive inclusion was employed. Visual-perceptual and/or visuospatial processing difficulties (Group “VIS”) were considered if there was a significant deficit (*p* < 0.05) on the Arrows test or the Matching geometrical patterns task. For patients not presenting the preceding profile, an executive functioning impairment (Group “EF”) was considered if *p* < 0.05 for at least one of the three executive tasks. Lastly, a weakness of the conceptual system (Group “KNOW”) was considered in case of failure (cut-off) on one of the proposed four tasks (Naming tools by function, Functional association, Knowledge of actions, Matching pantomime-tool). Patients who were not classified in one of the three groups VIS, EF, and KNOW were reported in the group “OTHERS.”

## Results

Results for each clinical group are summarized in **Table 4** as proportion of failure on each variable. Significant disturbances on gestures production were observed in 81% of patients (*n* = 22). Impairment on pantomime under verbal command was observed in 67% of patients.

**Table 4:** Proportion of failure for each variable across clinical groups.

	VIS (*n* = 8)		EF (*n* = 6)		KNOW (*n* = 4)		OTHERS (*n* = 9)		Total (*n* = 27)	
	*n*	%	*n*	%	*n*	%	*n*	%	*n*	%
**Conceptual tests (KNOW)**					
Naming tools by function	0	0	1	17	2	**50**	0	0	3	11
Functional association PEGV	2	25	0	0	1	25	0	0	3	11
Knowledge of actions	0	0	1	17	1	25	0	0	2	7
Matching pantomime-tool	0	0	1	17	0	0	0	0	1	4
**Representational gestures**					
Pantomimes – verbal instruction	7	**88**	4	**67**	3	**75**	4	44	18	**67**
Pantomimes – visual instruction	6	**75**	2	33	3	**75**	2	22	13	48
Symbolic gestures	2	25	3	**50**	0	0	1	11	6	22
**Imitation of meaningless postures**					
Finger configuration	5	**63**	3	**50**	3	**75**	1	11	12	44
One-hand configuration	4	**50**	2	33	2	**50**	0	0	8	30
Two-hands configuration	4	**50**	2	33	2	**50**	1	11	9	33
**Imitation of meaningless sequences**										
Finger sequencing task	7	**88**	1	17	3	**75**	3	33	14	**52**
Manual motor sequences NEPSY	7	**88**	3	**50**	1	25	0	0	11	41
**Real tool use**					
Multiple choice task	1	**13**	0	0	0	0	0	0	1	4
Multiple object task	0	0	0	0	0	0	0	0	0	0
**Visual-constructional skills (VSC)**					
Block design WISC-IV	6	**75**	0	**0**	1	25	3	33	10	37
Execution condition ROF	7	**88**	3	**50**	1	25	5	**56**	16	**59**
**Visual and visuospatial perception (VIS)**					
Arrows NESPY	7	**88**	0	0	0	0	0	0	7	26
Matching geometrical patterns PEGV	4	**50**	0	0	0	0	0	0	4	15
**Executive functions (EF)**					
Planning index ROF	2	25	2	33	0	0	0	0	4	15
Verbal fluidity	1	13	2	33	0	0	0	0	3	11
Stroop effect	3	38	4	**67**	0	0	0	0	7	26
**Intellectual efficiency and Comprehension**					
Matrix Reasoning WISC-IV	5	**63**	1	17	0	0	3	33	9	33
Vocabulary WISC-IV	4	**50**	1	17	0	0	0	0	5	19
Comprehension NEPSY	4	**50**	0	0	2	**50**	0	0	6	22

*Percentage of failure at or above 50% are marked in bold.*

The number of tasks failed by every patient on the various processing levels is detailed in **Table 5**. The analysis of the overall sample showed that there was at least one deficit on a representational gestural task in 74% of patients, on meaningless posture imitation in 52%, and on meaningless postures sequences in 63% of patients. Tasks assessing real tool use do not appear in this table because only one patient was significantly lower than TDs on the multiple-choice task (as indicated in **Table 4**).

**Table 5:** Number of failures within groups according to components and patients.

		KNOW	RG	NRG	MS	VSC	VIS	EF	Mat.	Voc.	Comp.
Patient	Group	(max = 4)	(max = 3)	(max = 3)	(max = 2)	(max = 2)	(max = 2)	(max = 3)			
P1	VIS	**1**	**3**	**3**	**2**	**2**	**2**	**3**	–	–	–
P2	VIS	**1**	**2**	**2**	**2**	**2**	**2**	0	+	–	–
P3	VIS	0	**2**	**3**	**2**	**2**	**2**	0	–	–	+
P4	VIS	0	**2**	**3**	**2**	**2**	**1**	0	–	–	–
P5	VIS	0	**3**	**1**	**2**	**2**	**1**	**1**	–	+	–
P6	VIS	0	0	0	0	0	**1**	0	+	+	+
P7	VIS	0	**1**	0	**2**	**2**	**1**	0	+	+	+
P8	VIS	0	**2**	**1**	**2**	**1**	**1**	**2**	–	+	+

P9	EF	**1**	**1**	0	**1**	0	0	**2**	+	–	+
P10	EF	0	**3**	**3**	**1**	**1**	0	**2**	+	+	+
P11	EF	0	0	0	0	0	0	**1**	+	+	+
P12	EF	0	**1**	0	**1**	**1**	0	**1**	+	+	+
P13	EF	**2**	**3**	**3**	0	**1**	0	**1**	–	+	+
P14	EF	0	**1**	**1**	**1**	0	0	**1**	+	+	+

P15	KNOW	**1**	0	**2**	**1**	0	0	0	+	+	+
P16	KNOW	**1**	**2**	**3**	**2**	0	0	0	+	+	–
P17	KNOW	**1**	**2**	0	0	**1**	0	0	+	+	+
P18	KNOW	**1**	**2**	**2**	**1**	**1**	0	0	+	+	–

P19	OTHERS	0	**1**	0	0	**2**	0	0	+	+	+
P20	OTHERS	0	0	0	0	**2**	0	0	–	+	+
P21	OTHERS	0	0	**1**	**1**	**1**	0	0	+	+	+
P22	OTHERS	0	**2**	**1**	**1**	**1**	0	0	–	+	+
P23	OTHERS	0	**1**	0	0	**1**	0	0	+	+	+
P24	OTHERS	0	0	0	0	**1**	0	0	–	+	+
P25	OTHERS	0	**2**	0	**1**	0	0	0	+	+	+
P26	OTHERS	0	**1**	0	0	0	0	0	+	+	+
P27	OTHERS	0	0	0	0	0	0	0	+	+	+

Total		30%	74%	52%	63%	67%	30%	33%	33%	19%	22%

*KNOW, conceptual tasks; RG, representational gestures; NRG, imitation of meaningless postures; MS, meaningless sequences; VSC, visual-constructional skills; VIS, visual and visuospatial perception; EF, executive functions; Mat., Matrix Reasoning; Voc., Vocabulary; Comp., Comprehension; max, maximum number of tasks that might be failed. For each patient, a failure (at least one) on the component is marked in bold.*

### Group DCD + VIS

One patient (P6) showed impairment on the Arrows subtest without any other significant failure on the overall study variables. Five patients (P1 to P5) shared deficits on Arrows subtest, overall visual-constructional measures, meaningless sequences imitation, pantomimes, imitation of finger configurations, and on at least one linguistic variable (Vocabulary and/or Comprehension). Only 2 of them were impaired on the symbolic gestures (P1 and P5) and/or on the Functional association task (P1 and P2). One of them was the only one to show difficulties on the multiple-choice task beside an overall executive dysfunction (P1). Common failures on meaningless sequences, pantomimes under verbal command and Execution condition of ROF was found in the remaining two patients (P7 and P8). One of them had also a lower performance on two out of three executive tasks (P8).

### Group DCD + EF

Executive disturbances mostly appeared in the Stroop task (patients P9 to P12). In general, the gestural profiles within the group “EF” showed heterogeneous performances. Nevertheless, a deficit on one task of meaningless sequences was observed in four out of the six patients (P9, P10, P12, and P14). Two of them (P9, P10) had impaired performances on two executive tasks including the Stroop. This group contains the only patient (P9) presenting a significant deficit on matching pantomime-tool, with a limited performance on pantomimes under verbal command whereas pantomimes under visual command and tool use tasks were preserved. In this patient, deficit on the Stroop test was associated with a significantly high planning index on the ROF. One patient (P10) showed an overall disturbance of gestural production, except for tasks of finger sequencing and real too use, and was also impaired on verbal fluidity.

Concerning the two patients with no significant failure on the meaningless sequences (P11, P13), the first one (P11) had no impairment at all except for executive functioning. The second patient (P13) was the only one presenting difficulties on two out of the four conceptual tasks (naming of tools and knowledge of actions). He also showed a significantly high planning index on the ROF, without being able to normalize the copy on the Execution condition. In addition, this patient had weak performance on representational gestures and meaningless gestures, but no deficit on tool use and linguistic tasks.

It is important to note that the “EF” group contains three out of the four patients who showed difficulties on the symbolic gestures (P10, P12, P13), without any deficit on linguistic variables.

Finally, the remaining patient (P14) presented a selective failure on verbal fluidity, associated with gestural disturbances limited to finger sequencing, pantomimes under verbal instruction, and imitation of finger configurations.

### Group DCD + KNOW

Four patients (P15 to P18) showed impairment on only one conceptual task without any perceptive or executive difficulties. No significant deficit was observed on tool use. One of them (P15) made a significant number of errors on naming tools by function, but without disturbance of representational gestures production. Two patients had difficulties on naming tools by function or functional association (P16 and P18, respectively) and shared deficits on the two pantomimes tasks, finger sequencing, imitation of finger and hand configurations (meaningless postures) as well as comprehension. Lastly, one patient (P17) performed poorly on knowledge of actions associated with an affection of the two pantomime tasks.

### Group DCD/Dyspraxia + OTHERS

As expected in view of the procedure employed to construct clinical groups, results for these nine remaining patients (P19 to P27) suggested the absence of perceptive visual/visuo-spatial, executive or conceptual deficits. Gestural performances (representational gestures, meaningless gestures, and meaningless sequences) were highly heterogeneous. Three patients (P20, P24, P27) showed no gestural deficit at all, and three others (P19, P23, P26) were impaired only on of pantomimes under verbal command. Six patients (P19 to P24) demonstrated a failure on at least one visual-constructional task, which was observed on the execution condition of the ROF for 5 of them (P19 to P23).

## Discussion

The present study aimed at determining the nature and the specificity of praxic impairment observed in DCD children. A theoretically guided examination was proposed in order to first address the different levels of processing that are involved in gestural production. This was made in reference to the cognitive models of apraxia (Roy and Square, 1985; Rothi et al., 1991), because the actual conception and assessment of praxic disorder in children has been fundamentally based on those models (Zoia et al., 2002). The contribution of other cognitive functions was also investigated. A multiple case study was conducted to allow discussing the theoretical predictions resulting from cognitive models, considering the important heterogeneity (inter and intra-individual) of the DCD.

### Praxis and Knowledge

In our sample of patients with DCD, gestural impairments frequently concerned representational gestures, in particular pantomimes under verbal command, for which deficits were observed in all groups. Failure proportion is notably lower when the same gestures were proposed under visual command. These results are in line with those reported in the literature (Dewey and Kaplan, 1992; Dewey, 1993; Hill, 1998; Zoia et al., 2002). From a theoretical perspective, this profile may raise the question of semantic or sensorimotor knowledge disturbance. In the present study, the semantic knowledge about tools and actions was assessed by means of three tasks. Thirty percent of patients showed an impaired semantic or sensorimotor knowledge, which was often limited to one test, and without associated perceptive and/or executive impairment in half of them (Group “KNOW”). Only one of them (P17) had a relatively “pure” profile, with an impairment of knowledge about actions associated with a selective deficit on the two tasks of pantomimes. However, this patient did not make any error on the tasks of real tool use, which necessitated choosing and associating tools/objects in a conventional way. So, in addition to the hypothesis of knowledge difficulties concerning the typical use, one should consider the assumption of a deficit on symbolization capacities, as suggested by Kaplan (1977). Indeed, contrary to the use of a tool (while holding it in hand), pantomimes necessitate selecting and representing the physical properties of the absent tool, which are useful for gesture recognition by others (Baumard et al., 2014). Furthermore, the communication of the idea of an action by means of an object may carry more temporo-spatial constraints than expressive symbolic gestures. This contributes to explain why the patient (P17) as well as most of our patients with a conceptual failure had no significant deficit on expressive symbolic gestures. Dewey and Kaplan (1992) also underlined the presence of more severe praxic disorders in children with motor deficits and receptive language impairment. As well, the anamnesis of P17 revealed a past history of speech difficulties despite the fact that he showed no significant weakness on Comprehension. This last task showed impaired performances in the two other patients of the group “KNOW” who had deficits on tasks of pantomimes, but also on meaningless sequences and imitation of meaningless postures. Although it can be contributory, language impairment involves only a small number of patients, and is not sufficient enough to explain the overall gestural disturbances observed.

The observation of representational gestures production has led some authors to suggest the hypothesis of a disorder relating to sensorimotor representations of previously learned actions (which would be stored in memory) (Zoia et al., 2002; Sinani et al., 2011), which directly reflect the idea of “gestural engram” in apraxia. In accordance with the critical analysis proposed by Baumard et al. (2014), this assumption presumes that sensorimotor knowledge links specific objects to specific movements. A deficit of this knowledge should disturb the production of all tasks of real or pantomime tool use. Although deficits on pantomimes were frequent in the cohort (67% under verbal command and 48% visual command), only one patient (P1) showed deficit on tool use. In line with the sensorimotor knowledge hypothesis, difficulties in pantomime recognition could be expected (Buxbaum, 2001). Only one patient showed such a deficit (P9) with a weak performance on pantomime under verbal command, but pantomime under visual command and real tool use were preserved. In addition, these two patients (P1, P9) showed impaired executive functioning (inhibition). Our results thus do not support the hypothesis of sensorimotor knowledge difficulties in DCD.

### Praxis, Visuoperceptive Disturbances, and Visuospatial Disorders

More than a conceptual disorder, gestural impairments observed in DCD could result from a production system deficit, in accordance with the fact that the observed failures were not limited to pantomimes, but also involved imitation of meaningless postures and sequences. This assumption corresponds better to the definition of DCD, which affects intentional motor activities (Vaivre-Douret, 2014). Regarding to the production level, the existence of a direct non-lexical route (Rothi et al., 1991) has been questioned by Goldenberg (2001), which predicted that visuospatial disorders should impact gestures imitation, and more particularly those performed under direct visual control (finger configurations). In line with this prediction, 60% of the patients of the group “VIS” (P1 to P5) showed impairments on imitation of finger configurations and weak scores on Arrows subtests (visuospatial processing), block design subtest, and the execution condition of the ROF. By contrast, our results did not show dissociation with hand configurations, which were also disturbed in the majority of these patients. Some methodological limitations could nevertheless be highlighted. Indeed, in our experimental design, meaningless postures were purposely matched with the representational gestures. The comparison between the different types of configurations is thus compromised because the hand configurations include certain degree of finger constraints, and vice versa. Besides, the dynamic nature of some meaningless gestures (contrary to static postures) provides an additional dimension to the imitation. Note that the difficulties of these patients also concerned the imitation of meaningless sequences and pantomimes. Two other patients in the group “VIS” (P7, P8) showed such difficulties, but no impairment on the imitation of postures. These differences tend to confirm the non-specificity of the praxic profile of those first patients (P1 to P5), because their gestural difficulties were inconstant. Beyond that, it could be considered that if defect on the visual analysis of the model to be imitated can be compensated by the topographic knowledge about body structure, a visuospatial disorder can compromise the feedback and the corrections of gestures in real time (Wilson and McKenzie, 1998; Tsai et al., 2008), in particular when the exerted dynamic constraints are important (as in pantomimes and the imitation of meaningless sequences). On another side, the intervention of oculomotor disorders that interfere with visual exploration could be discussed in the group “VIS,” which mainly involved failures in the subtest of Arrows. Indeed, this task requires that children visually judge the orientation of objects on the basis of visual exploration of the page. Langaas et al. (1998) suggested that oculomotor control disorders could be the expression of an overall deficit of motor coordination (involving different effectors), rather than the cause of these difficulties. This assumption, which has to be verified, sustains the debate since it was established that motor difficulties related to optic ataxia in adults must be differentiated from apraxia (Goldenberg, 2009).

### Praxis and Executive Functions

Concerning online motor control, studies also postulated the intervention of executive functions, especially inhibition skills (Wilson et al., 2012; Pratt et al., 2014; Ruddock et al., 2014). With reference to the work of Roy (1978), Cermak (1985) proposed the existence of a “primary planning dyspraxia” characterized by an inability to conceptually plan the sequence of required movements due to an executive disorder not limited to the motor sphere, which could result from a disruption of the frontal area. In accordance with the literature, executive disturbances were observed in some of our patients, and more often related to a deficit of inhibition on Stroop test. Although the majority of patients in the group “EF” (67%) had a significant deficit in imitation of meaningless sequences, the gestural profiles remained widely heterogeneous. Consequently, it has to be considered that the association between an executive dysfunction and gestural difficulties does not constitute a specific profile of DCD, in line with the proposal of Vaivre-Douret et al. (2011a). By contrast, the executive dysfunction in one of the patients of the group “EF” (P13) could be questioned. Indeed, he only showed a significantly high planning index on the ROF, whereas his performance remained significantly impaired despite the provided cues on the execution condition. Furthermore, the gestural difficulties of this patient were distinct from those of other patients of the group. He exhibited an impairment involving two conceptual tasks, as well as an overall deficit on representational gestures and meaningless postures imitation, but no significant failure on the imitation of meaningless sequences.

### Which Arguments for a Specific Praxic Disorder in DCD?

Among the 27 patients of the study, only 9 (33%) did not show any deficit on perceptive, executive, or conceptual tasks (P19 to P27). The absence of significant difficulty on praxis tasks (production) was observed in 3 of them (P20, P24, P27). According to the criteria of DCD subtypes suggested by Vaivre-Douret et al. (2011b), it is possible to consider a “visual-spatial/visual-constructional dyspraxia” in six patients (P19 to P24). More specifically, whereas the visual-spatial component (named visual-spatial-motor by the authors, addressed by the copy of figures) was evident in 5 of them (P19 to P23), the visual-constructional component (ability of 3D assembly) was identified in only 2 of those 5 patients (P19 and P20). For the authors, these profiles are related to disorder in the “visual-spatial motor integration” (p. 452) involved in movement programming. Surprisingly, those patients did not systematically show a significant gestural impairment, and if they did, difficulties were strongly heterogeneous. The pure praxic nature of the visual-constructional tasks (ROF, Block design) is thus questionable. In line with this, Rösblad (2011) suggested that this type of task does not able to determine the capacity of the child to use the visual information in the control of the action (e.g., object-directed). On another hand, the contribution of oculomotor impairments could be questioned, as underlined by Vaivre-Douret et al. (2011a). As seen in optic ataxia, those impairments could go with disruption of visuo-motor transformations. In such a case, the specificity of the gestural disorder should be discussed in line with the distinction made between optic ataxia and apraxia (Goldenberg, 2009).

### Limits

In the current study, the difficulty of identifying “pure” gestural disorders raises the question of methodological and/or statistical limits. Firstly, the criteria of deficits were probably more strict (modified *t*-test method of Crawford and Howell, 1998, with threshold *p* < 0.05; cut-off) than the limit commonly used (at least one, to one and a half standard deviations). On another hand, this could be due to a recruitment bias. Indeed, there was some delay between the established diagnoses and the inclusion procedure. It is thus possible that difficulties decreased thanks to an intervention program and/or, to the spontaneous reduction of maturational delay assumed in DCD (Hill et al., 1998; Zoia et al., 2002). The possibility of a bias due to the motives for consultation should also be considered. Indeed, school difficulties generally involved graphics that constitute a motor activity sensitive to many factors like perceptive (Rosenblum and Livneh-Zirinski, 2008) and executive (Rosenblum, 2013) skills. This is also the case for the clumsiness. It is thus possible that the diagnostic criterion of interference in everyday life associated with DCD diagnosis (criterion B, DSM-V) can favor the inclusion of other form of deficits non-specific to gestures.

## Conclusion

Our results underline the heterogeneity of DCD profiles, which are commonly reported in the literature, and consequently the need for proposing analyses that consider the individual variability. On the basis of the theoretical predictions resulting from cognitive models of apraxia and suggested hypotheses in children, the multiple case analysis demonstrated difficulties that mainly involve gestural production without identifying “pure” profiles of production disorder. Many patients had gestural disorders in association with other cognitive deficits (perceptive, comprehension, executive), in accordance with prior studies. Although the concomitant observation of these deficits does not necessarily involve a direct causal link (Wilson and McKenzie, 1998), their presence in an individual profile questions the specificity of praxic disorders.

Given that the hypothesis of sensorimotor knowledge disorder was not supported by the described profiles and that the pantomimes (representational transitive gestures) were non-specifically impaired within the different groups, the relevance of these tasks can be contested. The imitation of meaningless postures thus seems more useful for studying gestural production without any actual object. By contrast, the almost complete absence of real tool use impairment in our sample of patients with DCD represents an issue. Concerning the aspects of “production,” it should be noted that the quotation systems used in our study focused on the ability to demonstrate the habitual use of the tool, and not on the quality of motor manipulation. In addition, decrease of difficulties in the presence of the tool supposes that tool use requires other processes (i.e., other than knowledge, executive functions, motor skills), which are overlooked by the current assessment methods, and could be functional in DCD children. Consequently, it is necessary to propose examination conditions that allow identifying the children in which these others processes would be impaired. Studies about tool use, in adults (Osiurak et al., 2011) and children (Beck et al., 2011; Remigereau et al., 2016), currently offer such perspectives for further research.

## Author Contributions

OC conceived and designed the study, she was in charge of the collect, analysis and interpretation of the data, contributed to draft the work, reviewed and approved the final version and its submission. Her agreement has been accountable for all aspects of the work. CR contributed to the acquisition and the analysis of the data. She critically reviewed and approved the final version to be published and her agreement has been accountable for all aspects of the work. CF contributed to the acquisition of the data. She critically reviewed and approved the final version to be published and her agreement has been accountable for all aspects of the work. AR, DL, and SF participated to the conception of the study, critically reviewed and approved the final version to be published and their agreement has been accountable for all aspects of the work.

## Conflict of Interest Statement

The authors declare that the research was conducted in the absence of any commercial or financial relationships that could be construed as a potential conflict of interest.

## Footnotes

1 In France, a specific developmental disorder of motor function is often diagnosed under the term of dyspraxia. According to the EACD recommendations (Blank et al., 2012) “the term ‘dyspraxia’ has not become recognized as separate entity or subgroup of DCD” (p. 62). Therefore, this study included children with either a diagnosis of dyspraxia or DCD.
